# A neuropeptide signal confers ethanol state dependency during olfactory learning in *Caenorhabditis elegans*

**DOI:** 10.1073/pnas.2210462119

**Published:** 2022-11-07

**Authors:** Jonathan H. Lindsay, Laura D. Mathies, Andrew G. Davies, Jill C. Bettinger

**Affiliations:** ^a^Department of Pharmacology and Toxicology, Virginia Commonwealth University, Richmond, VA 23298;; ^b^VCU-Alcohol Research Center, Virginia Commonwealth University, Richmond, VA 23298

**Keywords:** ethanol, olfactory learning, state-dependent learning, neuropeptide

## Abstract

Alcohol intoxication can impact learning and this may contribute to the development of problematic alcohol use. In alcohol (ethanol)-induced state-dependent learning (SDL), information learned while an animal is intoxicated is recalled more effectively when the subject is tested while similarly intoxicated than if tested while not intoxicated. When *Caenorhabditis elegans* undergoes olfactory learning (OL) while intoxicated, the learning becomes state dependent such that recall of OL is only apparent if the animals are tested while intoxicated. We found that two genes known to be required for signal integration, the secreted peptide HEN-1 and its receptor tyrosine kinase, SCD-2, are required for SDL. Expression of *hen-1* in the ASER neuron and *scd-2* in the AIA neurons was sufficient for their functions in SDL. Optogenetic activation of ASER in the absence of ethanol during learning could confer ethanol state dependency, indicating that ASER activation is sufficient to signal ethanol intoxication to the OL circuit. To our surprise, ASER activation during testing did not substitute for ethanol intoxication, demonstrating that the effects of ethanol on learning and recall rely on distinct signals. Additionally, intoxication-state information could be added to already established OL, but state-dependent OL did not lose state information when the intoxication signal was removed. Finally, dopamine is required for state-dependent OL, and we found that the activation of ASER cannot bypass this requirement. Our findings provide a window into the modulation of learning by ethanol and suggest that ethanol acts to modify learning using mechanisms distinct from those used during memory access.

## Significance

An altered internal state experienced during learning can enhance the specificity of learned information and promote situation-specific recall. Intoxication during training can confer state dependency so that recall requires the same intoxication state. Olfactory learning (OL) in *Caenorhabditis elegans* can become state dependent during ethanol intoxication. A neuropeptide originating outside of the OL circuit activates a receptor tyrosine kinase within the circuit to signal intoxication during learning to confer state dependency. Surprisingly, intoxication is encoded using a distinct mechanism during recall. State information can be added to existing OL but cannot be removed from state-dependent OL. These observations provide insight into the modulation of learning by alcohol and demonstrate that the effects of intoxication on learning and memory are distinct.

Animals can modulate learning in response to differing internal and external environments. Adding contextual information to learning can enhance the diversity of available information and may allow animals to access situation-appropriate learned behaviors efficiently. State-dependent learning (SDL) can occur when information learned by an animal while it is in a particular altered internal state is most effectively recalled when the animal is tested in the same altered internal state. Drug intoxication–induced SDL is most effective, but other salient internal states such as pain and depressed mood can also confer state dependency to learning (1–5). SDL has been demonstrated in nematodes (6), goldfish (7), rodents (8, 9), dogs (10), nonhuman primates (11), and humans (12).

*Caenorhabditis elegans* uses chemosensation to respond to the environment and move toward attractive odors and away from repulsive odors (13–15). The response to olfactory cues is plastic; worms can diminish their chemotaxis response to an otherwise attractive odorant after a long exposure to that odorant in combination with food deprivation in a process of associative olfactory learning (OL; also called olfactory adaptation; 16–20). OL is revealed by a decrease in movement toward the attractive cue. We have previously shown that OL is subject to modulation by ethanol intoxication (6). When worms undergo OL while intoxicated with ethanol, the recall of OL becomes entirely dependent on the presence of a similar intoxicating dose of ethanol; this is SDL. We found that dopamine is required for SDL (6).

*C. elegans* SDL shares several important defining characteristics with mammalian SDL. First, SDL is asymmetric; learning that occurs while the animals are not intoxicated is recalled equally well when the animals are intoxicated and not intoxicated, whereas learning that occurs while the animals are intoxicated is more effectively recalled when the animals are similarly intoxicated. Second, state dependency can be overcome with overtraining. Third, state dependency requires a change in state, for example, subintoxicating concentrations of ethanol that act as odorant cues do not induce SDL in *C. elegans* (6).

Here, we have investigated SDL induced by ethanol intoxication in *C. elegans* to better understand how ethanol alters information processing. We extend previous studies to identify neurons that are outside of the characterized chemosensory neuronal circuit whose activity is required for generating state-dependent OL. We identify a molecular signal for ethanol intoxication that is used during learning to modify OL. We find that the neuropeptide HEN-1 acts in signaling ethanol intoxication, and the SCD-2 receptor tyrosine kinase, a known receptor for HEN-1, is required for SDL and can function in the AIA neuron. HEN-1 is expressed in the ASER chemosensory neuron, and activation of ASER is sufficient to signal ethanol intoxication during training even in the absence of ethanol. We show that dopamine is required in parallel or downstream of ASER activation for SDL. Surprisingly, ASER does not signal ethanol intoxication during testing, indicating that the state of intoxication is signaled by different mechanisms during learning and recall. Existing OL can have intoxication-state information added to render it state dependent, but state-dependent OL does not lose dependency on intoxication once SDL has been established, even if the ethanol signal is removed early in the process of OL. Our observations provide new insight into how ethanol influences neuronal signals to alter the acquisition and recall of a learned behavior.

## Results

**Fig. 1.**
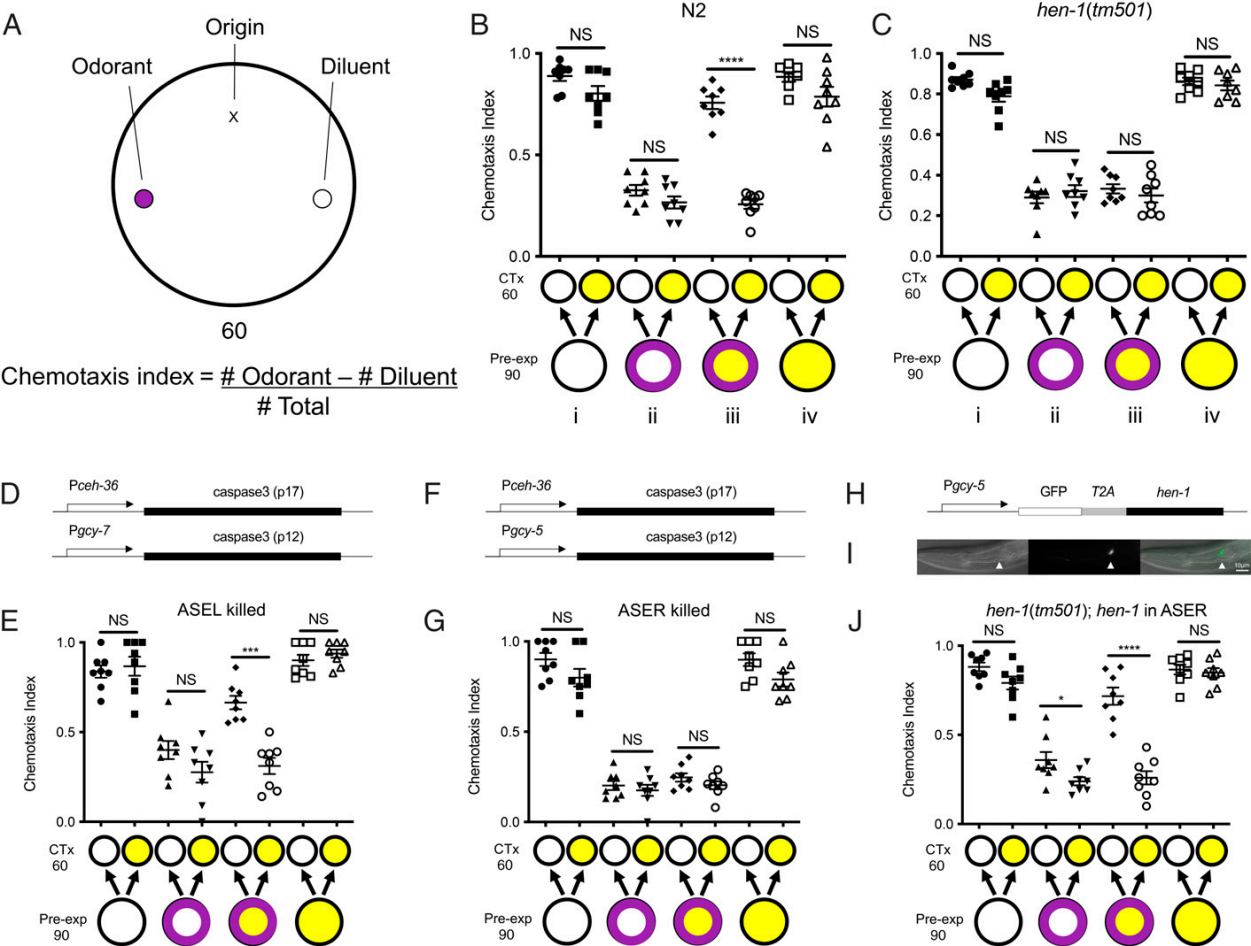
State-dependent OL assay; *hen-1* in ASER is sufficient for SDL. (*A*) In chemotaxis assays, a large plate is prepared with 2% agar and 1 µL of benzaldehyde (purple dot) and 1 µL of diluent (white dot). Worms (*n* = 50 to 100) are placed off-center and equidistant between the two spots (X) and allowed to move on the plate for 1 h before they are scored. Chemotaxis index = [number of animals at the odorant spot − number of animals at the diluent spot] / total number of worms in the assay. A chemotaxis index close to 1 indicates that the odorant is a strong attractant; a chemotaxis index close to 0 indicates that the odorant is not an attractant. (*B*) For the SDL behavioral assay, one large population of age-synchronized, first-day adult animals is divided onto four pre-exposure plates for 90 min (Pre-exp 90′): no odorant (*B, i*, white circle), benzaldehyde only (*B, ii*, white circle with purple ring), benzaldehyde plus 150 mM ethanol (*B, iii*, yellow circle with purple ring), and 150 mM ethanol only (*B, iv*, yellow circle). Each of these pre-exposure groups is washed off the plate and divided into two separate populations of 50 to 100 worms that are placed on paired chemotaxis assay plates for 60 min (CTx 60′): one plate contains no ethanol (white circle) and the other contains 150 mM ethanol (yellow circle). (*B, i*) Ethanol exposure during testing does not impair chemotaxis. (*B, ii*) Pre-exposure to benzaldehyde causes OL. Ethanol exposure during testing does not impair the demonstration of OL. (*B, iii*) Ethanol exposure during pretreatment with benzaldehyde confers state dependency on OL; animals only demonstrate OL when tested in the presence of ethanol. (*B, iv*) Prolonged ethanol pre-exposure does not alter chemotaxis. (*C*) *hen-1(tm501)*–null mutants do not learn state dependently; expression of OL that is learned during intoxication is not dependent on ethanol exposure during chemotaxis. (*C, i*) Ethanol exposure does not impair chemotaxis in *hen-1* mutants. (*C, ii*) *hen-1* mutants can perform OL; ethanol exposure during testing does not impair the recall of OL. (*C, iii*) Ethanol exposure during pretreatment with benzaldehyde does not confer state dependency on OL in *hen-1* mutants, indicating that *hen-1* is required for SDL. (*C, iv*) Prolonged ethanol pre-exposure does not alter chemotaxis in *hen-1* mutants. (*D*) Schematic of constructs that drive expression of the two caspase subunits in the OH8585 strain (22). Cells in which both subunits are expressed undergo programmed cell death. The caspase3 p17 subunit is expressed under the control of the *ceh-36* promoter, which is expressed in the ASE and AWC neuron pairs. The caspase3 p12 subunit is expressed under the control of the ASEL-specific *gcy-7* promoter. Both subunits are expressed only in ASEL. (*E*) Expression of caspase in ASEL does not impair SDL; expression of OL that is learned during intoxication is dependent on ethanol exposure during chemotaxis. (*F*) Schematic of constructs that drive expression of the two caspase subunits in the OH8593 strain (22). The caspase3 p17 subunit is expressed under the control of the *ceh-36* promoter, which is expressed in the ASE and AWC neuron pairs. The caspase3 p12 subunit is expressed under the control of the ASER-specific *gcy-5* promoter. Both subunits are expressed only in ASER. (*G*) Animals expressing caspase in ASER can undergo OL but do not learn state dependently; expression of OL that is learned during intoxication is not dependent on ethanol exposure during chemotaxis. (*H*) Schematic of the construct driving expression of *GFP::T2A::hen-1* under the control of the ASER-specific *gcy-5* promoter. (*I*) GFP expression in ASER in *hen-1(tm501); betEx3 [Pgcy-5::GFP::T2A::hen-1]* animals: (*Left*) Differential interference contrast microscopy (DIC) image of a young adult animal (exposure 50 ms), (*Middle*) fluorescent image (exposure 200 ms), (*Right*) fluorescent image overlay onto DIC image (white arrowhead indicates ASER). Anterior is to the *Left*. Scale bar, 10 µm. (*J*) Expression of *hen-1* in ASER in *hen-1(tm501)*–null mutants is sufficient to rescue the SDL defect of *hen-1(tm501)*; expression of OL that is learned during intoxication is dependent on ethanol exposure during chemotaxis. Error bars represent SEM. Statistical comparisons were made using unpaired multiple *t* tests (*n* = 8); bars indicate which datasets are being compared. *P < 0.05, ***P < 0.0001, ****P < 0.0001. CTx, chemotaxis; NS, not significantly different.

When *C. elegans* experience an extended exposure to a high concentration of an attractive olfactory cue in the absence of food, they decrease their attraction to that odorant in a process of associative OL in which the presence of the odorant and food deprivation are paired (16, 21). In this case, the odorant is likely to signal starvation rather than food, and therefore the response to the odor is attenuated, allowing the animals to escape an otherwise attractive cue. We quantify chemosensory response by exposing worms to an olfactory gradient and assessing their attraction to the odorant (in these studies, an attractive concentration of benzaldehyde) by calculating a chemotaxis index (Fig. 1 *A* and *B*, *i*; 16). When worms are exposed to benzaldehyde in the absence of food for 90 min, they subsequently decrease their attractive response to the odorant, which is reflected in a decrease in the chemotaxis index (Fig. 1 *B*, *ii*; 16).

We have previously demonstrated that OL can be made state dependent; when worms are exposed to intoxicating concentrations of ethanol during OL, they will only recall the OL if they are tested in the same intoxicated state, whereas similarly treated worms tested with no ethanol will show no evidence of having experienced the olfactory cue and will chemotax robustly toward the odorant (Fig. 1 *B*, *iii*, compare with Fig. 1 *B*, *ii*) (6). Ethanol exposure itself, either during chemotaxis or for a 90-min pre-exposure, or both, does not modify chemotaxis to benzaldehyde (Fig. 1 *B*, *i* and *iv*; 6).

True SDL should require a change in state. Ethanol acts as an olfactory cue for *C. elegans* (23); one possibility for the effect of ethanol on OL is that the animals are associating the smell of ethanol with benzaldehyde to create a complex cue, and this mimics true state dependency without requiring a change in state. We previously showed that low concentrations of ethanol that can act as olfactory cues but do not cause intoxication phenotypes do not induce SDL, indicating that a concentration-dependent property of ethanol induces SDL (6). To more carefully examine if the state-changing aspect of ethanol causes SDL, we tested a mutant strain that is resistant to intoxication. If intoxication is required for SDL, we would expect these animals to require higher concentrations of ethanol than would wild-type animals to induce SDL. The SLO-1 BK potassium channel is a direct target of ethanol and loss of *slo-1* results in strong resistance to ethanol intoxication (24). As predicted, we found that *slo-1(eg142)* null mutant animals did not demonstrate SDL at the same ethanol concentration (150 mM) that produces robust SDL in wild-type worms (*P* = 0.74; *t* = 0.81, degrees of freedom [df] = 14; *SI Appendix*, Fig. S1*A*). This defect in SDL can be overcome at a higher ethanol concentration (300 mM; *P* < 0.0001; *t* = 8.9, df = 14; *SI Appendix*, Fig. S1*B*), which is consistent with the observation that *slo-1* mutant animals can become intoxicated but at higher ethanol concentrations than wild-type animals (24). Together, these data strongly suggest that the state of ethanol intoxication is responsible for our observations of SDL.

We interpret SDL as being the product of the integration of at least three stimuli: the extended exposure to the olfactory cue in the absence of food (odor plus starvation [O+S]) that results in OL, and the intoxicating concentration of ethanol. To determine the mechanisms by which OL can be modulated by ethanol intoxication, we asked if genes that had been previously found to be required for processing multiple stimuli are also important for SDL. The secreted peptide HEN-1 is required by worms for the integration of different signals in several behavioral paradigms, including the decision whether or not to cross an aversive barrier to get to an attractive cue (25–28). We tested a null allele, *hen-1(tm501)*, in our SDL assay. The *hen-1* mutants were able to sense and navigate a gradient toward benzaldehyde in the presence or absence of an intoxicating concentration of ethanol (Fig. 1 *C*, *i*) and were able to demonstrate OL in the presence or absence of ethanol (Fig. 1 *C*, *ii*). Together, these results indicate that *hen-1* is not required for chemotaxis or OL to benzaldehyde. Ethanol alone during a 90-min pre-exposure period did not alter the ability of *hen-1* mutants to chemotax to benzaldehyde in the presence or absence of ethanol (Fig. 1 *C*, *iv*). However, *hen-1* mutant animals were completely unable to learn state dependently; they demonstrated decreased attraction to benzaldehyde (indicating OL) after a combination of ethanol and benzaldehyde pre-exposure, when tested either in the presence or absence of ethanol during chemotaxis (*P* = 0.53; *t* = 0.78, df = 14; Fig. 1 *C*, *iii*). This strongly suggests that HEN-1 is required for the integration of the different stimuli that result in the modulation of OL by ethanol intoxication.

HEN-1 is a secreted peptide. It is most highly expressed in ASER, but it is also expressed in AIY, RIR, AFD, RIC, AWC, RIS, AWA, ASI, ASK, BAG, PQR, and PVT (25, 29). We hypothesized that the likely source of the HEN-1 signal in SDL is the ASER neuron, because of its anatomical location; the ASE neurons synapse onto the AIA interneurons (30), which are known to be required for OL to benzaldehyde (31). The ASE neurons are a bilateral pair, ASER and ASEL, but of the two, only ASER neurons express *hen-1* (25). We examined the requirements for ASEL and ASER in SDL by using existing transgenic strains that express mammalian caspase specifically in each of these neurons to kill each cell individually (Fig. 1 *D* and *F*; 22). We found that in animals in which the ASEL neuron was killed, there was no effect on the ability to perform SDL (*P* = 0.0001; *t* = 6.01, df = 14; Fig. 1*E*). In contrast, animals in which the ASER neuron was killed were completely unable to learn state dependently (*P* = 0.54; *t* = 0.70, df = 14; Fig. 1*G*). This result indicates that the ASER, but not the ASEL, neuron is part of the circuitry underlying state dependency.

We hypothesized that ASER releases HEN-1 to confer state dependence on OL, so we next asked if HEN-1 expression in ASER was sufficient for generating state dependence by expressing *hen-1(+)* exclusively in ASER. We generated transgenic animals expressing *hen-1(+)* under the control of the ASER-specific *gcy-5* promoter in the *hen-1(tm501)* null mutant background (Fig. 1*H*). We coexpressed green fluorescent protein (GFP) from the same promoter and only assayed animals in which we could detect GFP expression in the ASER neuron (Fig. 1*I*). We found that expression of *hen-1* in ASER was sufficient for rescue of the SDL defect in *hen-1* null mutants (*P* < 0.0001; *t* = 7.51, df = 14; Fig. 1*J*). Together, these observations support a model in which ASER confers ethanol-induced state dependency using a HEN-1 signal.

**Fig. 2.**
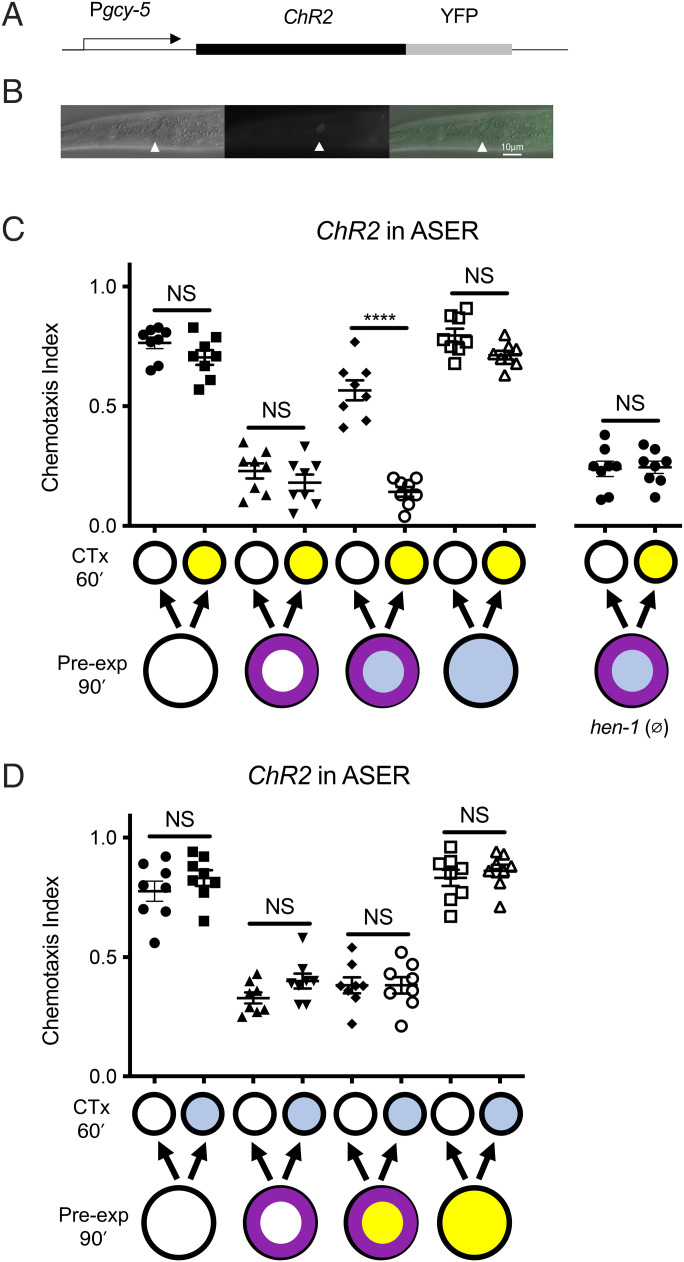
Optogenetic activation of the ASER neuron during training but not during testing substitutes for ethanol and confers state dependency to OL. (*A*) Schematic of the construct driving expression of *ChR2*(*H134R)::YFP* under the control of the ASER-specific *gcy-5* promoter. (*B*) YFP expression in ASER in *betEx12* [*Pgcy-5::ChR2::YFP*] animals: (*Left*) DIC image of a young adult animal (exposure 50 ms), (*Middle*) fluorescent image (exposure 200 ms), (*Right*) fluorescent overlay onto the DIC image (white arrowhead indicates ASER). Anterior is to the *Left*. Scale bar, 10 µm. (*C*) Optogenetic activation of ASER during pre-exposure to benzaldehyde confers state dependency to OL in the absence of ethanol; expression of OL that is learned while ASER is activated is dependent on ethanol exposure during chemotaxis. In contrast, optogenetic activation of ASER during pre-exposure in *hen-1* null mutants carrying *betEx12* did not confer state dependency to OL. (*D*) Optogenetic activation of ASER during testing does not replace ethanol exposure in SDL; OL that is learned during intoxication is not expressed in the absence of intoxication if ASER is activated during chemotaxis. On the x-axis, pale blue, filled circle indicates blue light exposure; purple ring indicates benzaldehyde pre-exposure; yellow indicates ethanol exposure. Error bars represent SEM. Statistical comparisons were made using unpaired multiple *t* tests except for the *hen-1* experiment, which used an unpaired *t* test (*n* = 8); bars indicate which datasets are being compared. ****P < 0.0001. CTx, chemotaxis; NS not significantly different.

*hen-1* function is required for several sensory integration paradigms (25–27). One possibility is that release of HEN-1 is permissive for the modulation of learning; in such a case, *hen-1* function would be required but not sufficient for SDL. In contrast, if a HEN-1 signal from ASER constitutes the ethanol intoxication signal, we would expect that activating ASER in the absence of ethanol would be sufficient to confer state dependency. We used channelrhodopsin to selectively activate the ASER neuron (32, 33). We generated transgenic animals expressing channelrhodopsin under the control of the ASER-specific *gcy-5* promoter (Fig. 2 *A* and *B*) and used blue light to activate channelrhodopsin during OL to ask if the activation of ASER was sufficient to confer state dependency. Channelrhodopsin function requires all-trans retinol (ATR), which is provided in the diet. In animals that had not been fed ATR, treatment with light during learning did not affect OL (*P* = 0.17; *t* = 1.72, df = 14; *SI Appendix*, Fig. S2). In contrast, we found that in animals fed ATR, ASER activation in the absence of ethanol was sufficient to confer state dependence to OL; when these animals were tested, they demonstrated OL only in the presence of ethanol (*P* < 0.0001; *t* = 9.126, df = 14; Fig. 2*C*). This result indicates that activation of ASER confers state dependency to OL. Our model is that ethanol intoxication during OL is signaled by the release of HEN-1 from ASER, and this signal modulates the encoding of OL to confer state dependency.

If the optogenetic activation of ASER causes the release of HEN-1, which is required for SDL, then we would expect that loss of *hen-1* would prevent activation of ASER from generating SDL. We tested this hypothesis by optogenetically activating ASER in the *hen-1(tm501)* null mutant background. As predicted, we found that ASER requires functional *hen-1* to generate state dependency; activation of ASER in the *hen-1* mutant background during learning could not confer state dependency to OL (*P* = 0.88; *t* = 0.15, df = 14; Fig. 2*C*). These data suggest that HEN-1 is released from ASER to confer state dependency, although this result is also consistent with a requirement for *hen-1* that is downstream or in parallel to ASER activation.

To demonstrate SDL, animals must recognize and signal ethanol intoxication both during training (causing OL to be associated with intoxication) and during testing (signaling that the animals are in the same intoxicated state during the chemotaxis assay). The simplest model predicts that the ethanol intoxication signal would be the same in both phases of the assay, so we tested if ASER activation could mimic ethanol intoxication during both training and testing. To our surprise, when we replaced ethanol intoxication by ASER activation during both training and testing, we did not observe state dependency in OL (*P* = 0.98; *t* = 1.25, df = 14; *SI Appendix*, Fig. S3). Given that ASER activation during pre-exposure was sufficient to mimic ethanol intoxication (Fig. 2*C*), this strongly suggested that ASER activation during testing could not provide the intoxication signal. To directly test ASER’s role during the recall of state-dependent OL, we performed an assay in which the animals were exposed to benzaldehyde in the presence of ethanol and then tested in the absence of ethanol with and without optogenetic activation of ASER. We found that ASER activation during testing could not substitute for ethanol intoxication in the recall of state-dependent OL (*P* > 0.9999; *t* = 0, df = 14; Fig. 2*D*). Together, these results point to a more complicated model for state dependency where ASER activation is only sufficient for signaling ethanol intoxication during training and a different ethanol signal is used during testing.

**Fig. 3.**
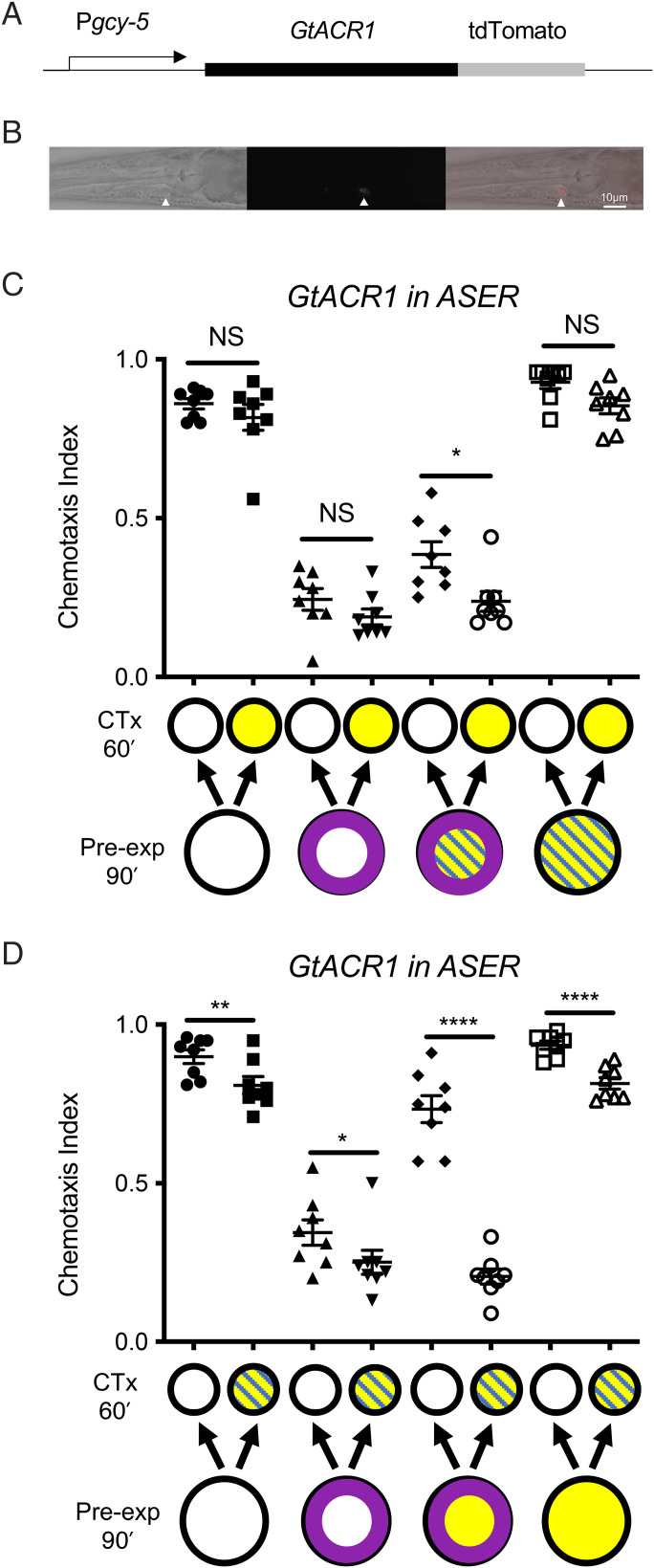
Optogenetic inhibition of ASER during training, but not during testing, prevents SDL, indicating that the ethanol intoxication signals in learning and recall are distinct. (*A*) Schematic of the construct driving expression of *GtACR1::tdTomato* using the ASER-specific *gcy-5* promoter. GtACR1 encodes anionic channelrhodopsin. (*B*) tdTomato expression in *betEx14* [*Pgcy-5::GtACR1::tdTomato]* animals: (*Left*) DIC image of a young adult animal (exposure 50 ms), (*Middle*) fluorescent image (exposure 200 ms), (*Right*) fluorescent overlay onto the DIC image (white arrowhead indicates ASER). Anterior is to the *Left*. Scale bar, 10 µm. (*C*) Optogenetic inactivation of ASER during learning prevents ethanol intoxication from conferring state dependency on OL, supporting the observation that ASER is required for the intoxication signal during learning. (*D*) Optogenetic inactivation of ASER during testing does not interfere with SDL, indicating that ASER activity is not necessary for the ethanol signal during recall of state-dependent OL. Diagonal blue lines represent blue light exposure; purple ring indicates benzaldehyde pre-exposure; yellow indicates ethanol exposure. Error bars represent SEM. Statistical comparisons were made using unpaired multiple *t* tests (*n* = 8); bars indicate which datasets are being compared. *P < 0.05, **P < 0.01, ****P < 0.0001. CTx, chemotaxis; NS not significantly different.

One possibility is that ASER activation is required during both training and testing to signal intoxication, but that an additional ethanol-induced signal is also required during testing. To further probe the differences in the intoxication signal in training and testing, we generated animals in which we could optogenetically inactivate ASER. We generated animals expressing the anionic channelrhodopsin GtACR1 under the control of the ASER-specific *gcy-5* promoter (Fig. 3 *A* and *B*). Our model suggests that inactivation of ASER during learning should not affect normal OL but should prevent the development of SDL. Indeed, we found that optogenetic inhibition of ASER during learning strongly reduced SDL (*P* = 0.037; *t* = 2.89, df = 14; Fig. 3*C*), consistent with our earlier observations in animals in which ASER was killed (Fig. 1*G*). We next asked if ASER signaling contributes to the intoxication signal during recall of SDL. We inactivated ASER during testing and found that the animals were, nevertheless, able to demonstrate state-dependent recall (*P* < 0.0001; *t* = 10.80, df = 14; Fig. 3*D*). We noted that inactivation of ASER during testing resulted in a small but significant decrease in chemotaxis to benzaldehyde; this may reflect a small but important role for ASER in basal chemotaxis behavior. These results strongly support a model in which ethanol intoxication is encoded using distinct and nonoverlapping mechanisms during learning and recall.

**Fig. 4.**
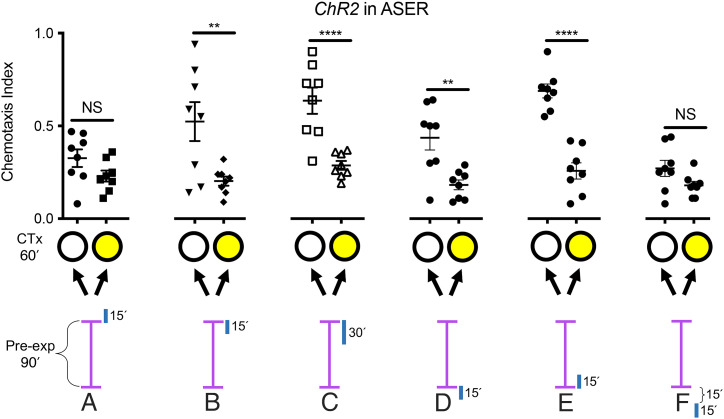
ASER activation and the olfactory cue and food deprivation must be presented together for SDL; OL can be converted to SDL, but SDL does not lose state information. *betEx12* [*Pgcy-5::ChR2::YFP*] animals are pre-exposed to benzaldehyde for 90 min (purple vertical line), with various shorter exposures to blue light. Short blue vertical lines indicate the timing and duration of light pulses; yellow indicates ethanol exposure. (*A*) Activation of ASER immediately following benzaldehyde exposure does not confer state dependency on OL. (*B*) Activation of ASER in the final 15 min of benzaldehyde pre-exposure causes OL to become partially state dependent. (*C*) Activation of ASER in the final 30 min of benzaldehyde pre-exposure confers stronger state dependency on OL. (*D*) Activation of ASER for 15 min prior to benzaldehyde pre-exposure causes OL to be partially state dependent, despite the absence of ASER activation for the entire benzaldehyde pre-exposure. Optogenetic activation of ASER may cause release of supraphysiological levels of the intoxication signal, possibly HEN-1, so that the beginning of the exposure to benzaldehyde may be temporally coincident with the intoxication signal. (*E*) Activation of ASER during the first 15 min of benzaldehyde pre-exposure confers strong state dependency on OL. (*F*) Activation of ASER for 15 min followed by a 15-min rest interval to allow dissipation of a potential supraphysiological intoxication signal before benzaldehyde pre-exposure does not cause SDL. Error bars represent SEM. Statistical comparisons were made using unpaired *t* tests (*n* = 8); bars indicate which datasets are being compared. **P < 0.01, ****P < 0.0001. CTx, chemotaxis; NS not significantly different.

Our interpretation of these observations is that the animals associate the state of ethanol intoxication with the O+S signals that underlie OL. We wondered about the kinetics of this association and if the ethanol stimulus had to be coincident with OL. We previously found that sequential OL (90 min) followed by ethanol exposure (20 min) did not confer state dependency to OL (6), but those experiments may have been somewhat confounded by the time that it takes for ethanol to accumulate to an intoxicating concentration. Because optogenetic activation of ASER can confer state dependency to OL, we could probe the timing requirements for the ethanol signal to confer state dependency at a high temporal resolution. We began by asking if we could make existing OL state dependent by activating ASER immediately after training. We found that, consistent with our previous observations (6), this sequential presentation of O+S followed by ASER activation was insufficient to modify OL to become state dependent (*P* = 0.11; *t* = 1.73, df = 14; Fig. 4*A*). This result suggests that the temporal coincidence between the signals is important.

We next asked if we could modify existing OL by adding ASER activation after learning had already been established but while exposure to O+S was ongoing. We allowed the animals to experience benzaldehyde for 75 min and then added ASER activation to the O+S presentation for 15 min and observed an intermediate result. While the animals showed some evidence of OL in the absence of ethanol, the degree of learning appeared to be decreased (*P* < 0.01; *t* = 2.98, df = 14; Fig. 4*B*), suggesting that conversion of existing OL to state dependency may be possible. We then extended the interval of coincident benzaldehyde exposure and ASER activation to 30 min. We found that if we allowed the animals to establish OL for 60 min and then added ASER activation for the last 30 min of O+S presentation, we could convert OL to state dependence (*P* < 0.001; *t* = 4.66, df = 14; Fig. 4*C*). This result suggests that there is plasticity to the OL even after it is established, and that the addition of new state information (intoxication) can modify existing learning.

Next, we tested if activation of ASER before OL could confer state dependency. We found that a 15-min activation of ASER followed by 90 min of O+S gave what appeared to be an intermediate phenotype; there was evidence that the recall of OL had developed some ethanol dependence, but it was not completely state dependent (*P* < 0.01; *t* = 3.59, df = 14; Fig. 4*D*). This is in contrast to our previous observation that sequential exposure to 20 min of ethanol intoxication followed by 90 min of OL was insufficient to confer state dependency (6). One possible explanation for these observations is that ASER activation has a significant and lasting effect on OL due to the extremely strong nature of optogenetic activation. We hypothesize that supraphysiological levels of HEN-1 are released during optogenetic activation, and residual HEN-1 may still be present when OL begins, which would be temporally coincident with learning and may therefore signal state dependency.

If this model is correct, it suggests that brief exposure to ASER activation at the beginning of OL should be sufficient to confer state dependency, and state dependency should then persist even after removal from the ASER signal. We therefore asked if activation of ASER during only the first 15 min of OL was sufficient to confer state dependency. We found that 15 min of ASER activation could confer state dependency to OL and that state dependency was long lasting (*P* < 0.0001; *t* = 7.48, df = 14; Fig. 4*E*). We noted that our model of supraphysiological release of HEN-1 due to ASER activation predicts that the actual exposure to HEN-1 would extend beyond the 15 min of ASER activation. To carefully test how long after ASER activation OL could be modified, we activated ASER for 15 min, then waited 15 min before beginning OL. With this exposure paradigm, we found that we did not generate SDL (*P* = 0.081; *t* = 1.88, df = 14; Fig. 4*F*). We interpret these findings to suggest that the additional 15 min of rest resolves lingering HEN-1, preventing temporal coincidence of HEN-1 signals with OL signals.

**Fig. 5.**
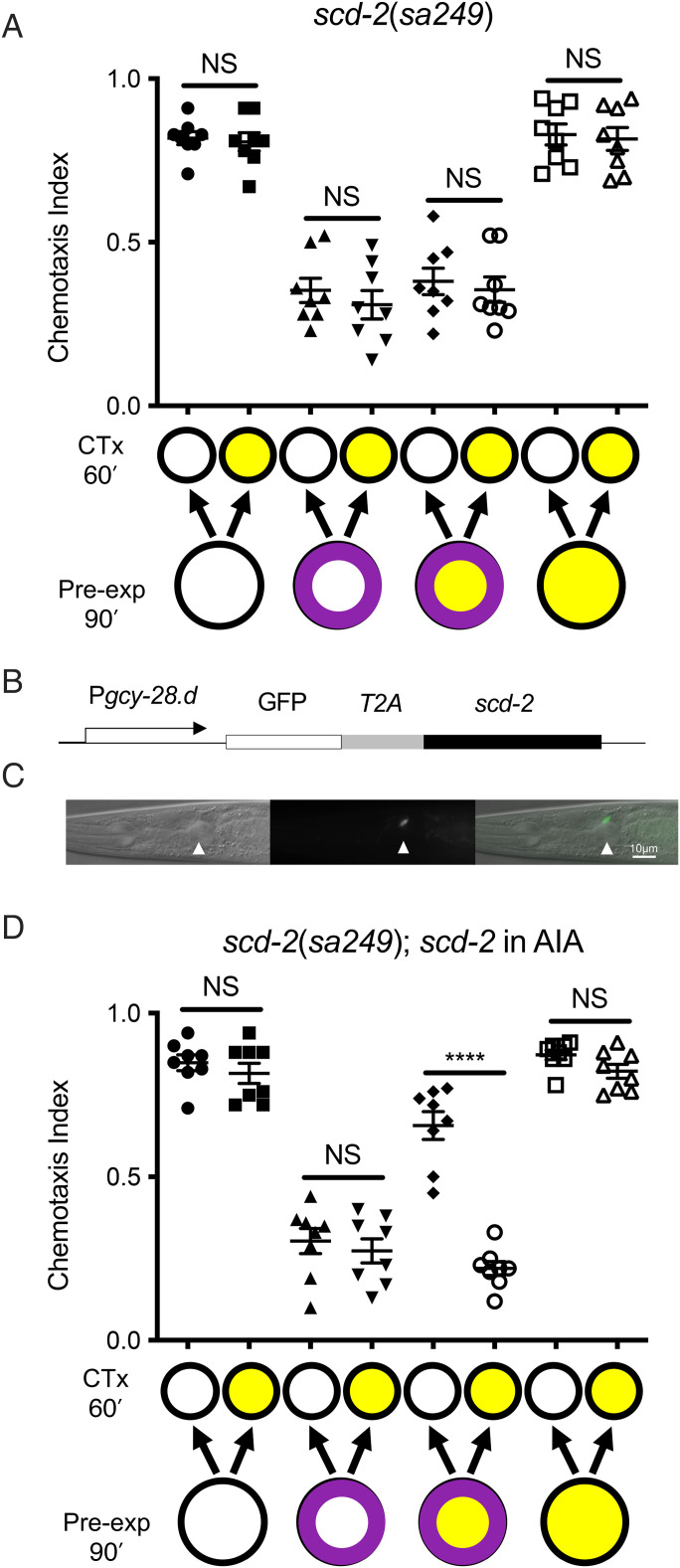
*scd-2* expression only in AIA interneurons is sufficient for SDL. (*A*) *scd-2(sa249)*–null mutant animals do not learn state dependently; OL that is learned during intoxication is not dependent on ethanol exposure during testing. (*B*) Schematic of the construct driving expression of *GFP::T2A::scd-2* under the control of the AIA-specific *gcy-28.d* promoter. (*C*) GFP expression in JCB300: *scd-2(sa249); betEx8 [Pgcy-28.d::GFP::T2A::scd-2]* animals: (*Left*) DIC image (*Middle*) fluorescent image (exposure 300 ms) (*Right*) fluorescent image overlay onto DIC image (white arrowhead indicates AIA). Anterior is to the *Left*. Scale bar, 10 µm. (*D*) Expression of *scd-2* in AIA neurons is sufficient to rescue the SDL defect of *scd-2(sa249)* mutants. Purple ring indicates benzaldehyde pre-exposure; yellow indicates ethanol exposure. Error bars represent SEM. Statistical comparisons were made using unpaired multiple *t* tests (*n* = 8); bars indicate which datasets are being compared. ****P < 0.0001. CTx, chemotaxis; NS not significantly different.

The secreted peptide HEN-1 binds to and activates the SCD-2 receptor tyrosine kinase, and *scd-2* function is required for the integration of sensory stimuli in different behavioral paradigms (28, 34–36). We predicted that SCD-2 activation would be required for state dependency. We performed an SDL assay on *scd-2(sa249)* null mutant animals and found that, while these animals were able to perform OL, they were unable to learn state dependently (*P* = 0.82; *t* = 0.45, df = 14; Fig. 5*A*). Thus, the defects in the *scd-2* animals are specific to the ability to modulate OL state dependently. Taken together, this suggests a model in which the HEN-1 signal acts on the SCD-2 receptor to promote state dependency.

*scd-2* is expressed in several neurons, and it is required in AIA interneurons for the ability to integrate multiple stimuli to make a choice between conflicting cues of an attractive and a repulsive stimulus (28). AIA neurons are part of the OL circuit for benzaldehyde (20, 31), suggesting that AIA is an excellent candidate for receiving the HEN-1 signal. We asked if *scd-2* functions in AIA neurons for SDL by testing a strain in which we provided *scd-2* expression under the control of the *gcy-28.d* AIA-specific promoter (28) in an otherwise *scd-2* null mutant background (Fig. 5 *B* and *C*). We found that expression of *scd-2(+)* in AIA was sufficient to rescue the SDL defect of *scd-2(sa249)* (*P* < 0.0001; *t* = 9.16, df = 14; Fig. 5*D*). Together, our data demonstrate that ASER and AIA are components of the SDL circuitry, and the molecular signaling pathway using HEN-1 and SCD-2 is required for SDL.

**Fig. 6.**
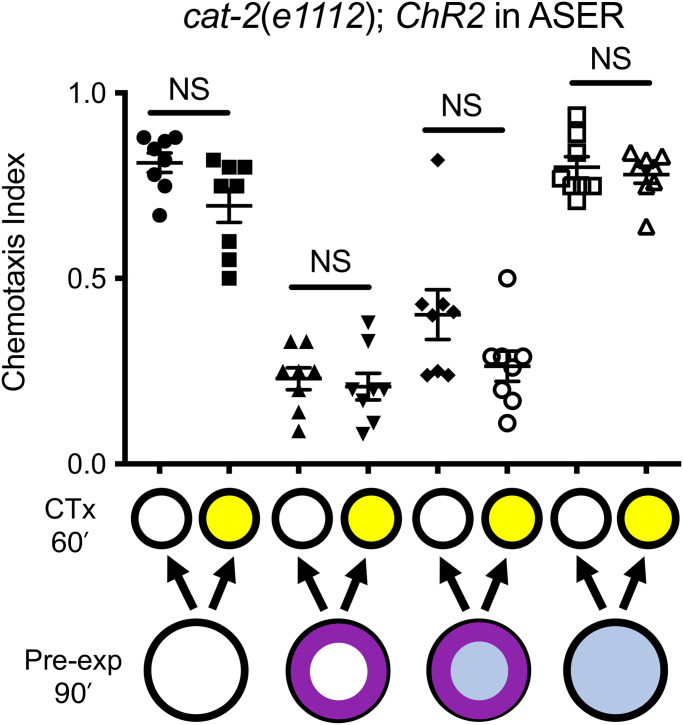
ASER activation cannot bypass the requirement for *cat-2* in SDL. *cat-2* mutants, which lack dopamine, do not learn state dependently (6). *cat-2* mutants carrying the *betEx12* construct expressing *ChR2(H134R)::YFP* in ASER can perform OL but do not learn state dependently when ASER is optogenetically activated during learning. Pale blue filled circles represent blue light exposure; purple ring indicates benzaldehyde pre-exposure; yellow indicates ethanol exposure. Error bars represent SEM. Statistical comparisons were made using unpaired multiple *t* tests (*n* = 8); bars indicate which datasets are being compared. CTx, chemotaxis; NS not significantly different.

Previously, we demonstrated that dopamine signaling is required for SDL; *cat-2* mutant animals that are unable to synthesize dopamine cannot learn state dependently (6). We asked if the dopamine signal is upstream of ASER activation by testing if activation of ASER during learning could bypass the requirement for dopamine in SDL. We crossed our ASER-specific channelrhodopsin construct into the *cat-2(e1112)* null mutant background and found that ASER activation during learning was unable to cause state dependency in these animals (*P* = 0.20; *t* = 1.76, df = 14; Fig. 6). The ChR2 construct is expressed in the *cat-2* mutant background (*SI Appendix*, Fig. S4*A*), and exogenous dopamine treatment restored the ability of ASER activation to confer state dependency to learning in these animals (*P* < 0.001; *t* = 4.99, df = 14; *SI Appendix*, Fig. S4*B*). These results indicate that the requirement for dopamine in SDL is downstream or parallel to ASER activation.

## Discussion

In *C. elegans*, ethanol intoxication can confer state dependency on OL (6). OL is an associative learned behavior that occurs when prolonged exposure to a high concentration of an attractive odor (the conditioned stimulus) in the absence of food (the unconditioned stimulus) reduces the response to a subsequent exposure to the same odor. Here, we show that ethanol intoxication signals to the OL circuit through activation of the ASER neuron during OL to confer ethanol state dependency on OL. Our data support a model in which ASER signals the OL circuit via a HEN-1 signal that is received by AIA neurons using the SCD-2 receptor.

The HEN-1 peptide is required for SDL. It is expressed in several neurons, including the benzaldehyde-sensing neuron AWC (25, 29), but it is most highly expressed in ASER. HEN-1 is secreted, so its anatomical origin in SDL may be difficult to define because release from any of several local neurons may be sufficient for its function. We considered the ASER neuron to be a good candidate for the locus of *hen-1* function in SDL because of the requirement for ASER in SDL, and because ASER synapses onto the AIA neurons, which are part of the known OL circuit. We found that HEN-1 expression in ASER is sufficient for SDL, and we suggest that HEN-1 acts as the intoxication signal from ASER that modifies OL during learning. HEN-1 signals via the SCD-2 receptor tyrosine kinase, which functions in the AIA neurons for SDL. Optogenetic activation of ASER in the absence of ethanol is sufficient to signal ethanol intoxication during learning, indicating that this is how ethanol intoxication feeds into and modifies OL.

We found that OL and ASER activation must be temporally coincident to confer state dependency. This suggests that the mechanism by which OL becomes state dependent requires incidence detection, and this may point to specific processes underlying OL that can be reinforced by intoxication. The ability to detect precisely overlapping stimuli is important for the ability of animals to be able to execute situation-specific behaviors. We found that while information about intoxication state can be added to existing OL, such information cannot be removed once it is established, suggesting that OL remains plastic even after it is established, but that plasticity is lost when OL is state dependent.

ASER activation is unable to confer state dependency in dopamine-deficient mutants. This result is consistent with at least two possibilities: There may be a requirement for dopamine during training that acts downstream of or in parallel with ASER activation. Alternatively, dopamine may participate in the process of recall, so that in these experiments, ASER activation may indeed induce state dependency, but we are unable to detect it because the animals do not recognize the intoxication state during testing.

Intriguingly, while ASER activation signals ethanol intoxication during training, ASER activation during testing does not substitute for ethanol intoxication. This indicates that the mechanism by which intoxication is signaled is different during learning and recall. Our data are consistent with a model in which signaling via HEN-1 and SCD-2 sets up a cellular state that indicates intoxication, and a separate molecular signal that is responsive to the presence of ethanol is involved in the recall of that state. We do not yet know what signals intoxication during recall, but one possibility is that dopamine is required in this process.

The activity of ASER itself is affected by ethanol exposure (37). ASER is a sensory neuron that is responsive to changes in NaCl concentration, it is acutely inactivated by increases, and activated by decreases in NaCl (38). Wang et al. (37) examined the effects of ethanol on the ASER response to NaCl as reported by calcium imaging. Somewhat surprisingly, ethanol suppressed the decrease in calcium release in ASER in response to up-steps of NaCl concentration but did not affect calcium release in response to down-steps of NaCl concentration (37), suggesting that ethanol suppresses inactivation but not activation of ASER. Our studies suggest that ethanol intoxication stimulates the release of the intoxication signal from ASER. We do not yet know if this action of ethanol is directly on ASER, or if ASER receives a signal that is induced by ethanol. Given the observation that ethanol can alter ASER function, one possibility is that ethanol suppresses the inhibition of the release of a signal such as HEN-1. Release of the signal provides contextual (in this case, state) information to the OL machinery.

### Integration of State Information with the Molecular Mechanisms of OL.

At least some of the molecular mechanisms of OL have been well defined, and these suggest that OL requires some changes to the function of the AWC chemosensory neurons that are likely to be long lasting. The translocation of the cyclic guanosine monophosphate–dependent protein kinase EGL-4 to the nucleus of AWC neurons is required for OL to AWC-sensed odorants, but the actions of EGL-4 in the nucleus are not yet understood (19, 20, 39, 40). The transcription factor SDF-13/TBX-2 is also required for OL (41), although it is localized primarily to the cytoplasm, suggesting a model in which it may be translocated with EGL-4 to the nucleus to help regulate transcription.

The AIA neurons are also required for OL; together, AIA and AWC make up the OL circuit. AIA activity during learning is required for EGL-4 translocation in AWC (20). There is a neuropeptide feedback loop that causes transient suppression of calcium signals in both AWC and AIA during learning (31); this reinforces the translocation of EGL-4 into the nucleus in AWC (20, 42–45).

Signals from outside of the AWC-AIA circuit can inhibit or enhance OL. Food deprivation is required for OL (46, 47), and this signal is likely to enter the circuit via an INS-1 insulin signal from ASI or AIA (48). The developmental experience of animals can influence the degree of OL; animals that experience crowding during development demonstrate stronger decreases in response to the odorant (49). This requires the SNET-1 signal, possibly transduced through the AIM neuron (49). Here, we demonstrate that ethanol intoxication can use a signal from ASER to modify OL in a way that is different from simple inhibition or enhancement of learning.

Because ethanol intoxication causes expression of OL to be conditional, and because the behavior manifests immediately after the animals are removed from their training conditions, it is difficult to reconcile the effects of intoxication on OL with a mechanism that causes long-term decreases in AWC function in response to odorant exposure. If state-dependent OL shares the same basic molecular mechanisms as non–state-dependent OL, how is it that this relatively long-term cellular effect (OL) rapidly diminishes when ethanol intoxication is not presented during testing? We have previously shown that a 35-min period of nonintoxication between pre-exposure and testing does not eliminate the state dependency of OL (6), so the removal of ethanol from the environment for this length of time is insufficient to diminish state-dependent OL. It will be interesting to examine the known mechanisms of OL for roles in SDL to determine if there is a shared mechanism for OL in state-dependent and non–state-dependent OL, and if, or how quickly, some of these mechanisms might revert in nonintoxicating conditions.

### SDL in Drug Dependence.

An important cue in drug-related learning is the intoxicating or state-altering property of the drug itself. Significant state dependency can be demonstrated in at least two fundamental physiological aspects of addiction: sensitization and tolerance. Behavioral sensitization to drugs can be state dependent; mice exposed to amphetamine and chlordiazepoxide for 8 d subsequently only demonstrated amphetamine sensitization when they were exposed to amphetamine in combination with chlordiazepoxide, not when they were exposed to amphetamine alone (50). Drug tolerance can also be made state dependent. Rats given morphine for 5 d developed substantial tolerance to the analgesic effects of the drug; however, expression of this tolerance could be blocked if the animals were tested with morphine while intoxicated by pentobarbital. In contrast, in rats in which the morphine training occurred in the presence of pentobarbital, tolerance to morphine’s analgesic effects was observed when the animals were tested with morphine while intoxicated with pentobarbital (51).

SDL is also thought to be important in promoting drug seeking. Individuals who used cocaine chronically showed state-dependent changes in dopamine signaling in the striatum during a probabilistic loss-learning task (52), which predicted an increase in the desire to use cocaine. SDL may also contribute to alcohol seeking because memories of negative consequences of intoxication are not easily accessible during abstinence from the drug; state dependency may explain in part why negative consequences of heavy drinking may not influence the decision to start drinking again when sober (53).

Dopamine plays a major role in the overlap of drug-conditioned contexts with reward circuitry of the mesolimbic system (54–57). Notably, dopaminergic neurons originating from the ventral tegmental area that project to the nucleus accumbens become activated in response to conditioned stimuli associated with alcohol seeking in rats (56). Chemogenetic inhibition of these neurons blocks alcohol-seeking behaviors even in the presence of conditioned alcohol cues (56). Dopamine is important for SDL in mammals (58–60) and is required for ethanol-induced SDL in *C. elegans* (6), and our data support a model in which dopamine may be involved during the recall phase of state dependency.

As we better understand how state is layered upon learning and how learning mechanisms can be modified by passages through altered states, we will gain insight into how alcohol may impact problematic behaviors such as drug seeking and relapse. Identifying the mechanisms by which contexts become tied to and influence drug use may assist in the development of novel treatment options for addiction.

## Materials and Methods

### *C. elegans* Strains and Husbandry.

*C. elegans* strains were maintained on lawns of *Escherichia coli* strain OP50 on nematode growth medium at 20 °C (61). Strains used in this study are listed in *SI Appendix, SI Materials and Methods*. All animals used in behavioral assays were age-matched, first-day adult hermaphrodites that had been reared for at least two generations in uncrowded, well-fed conditions.

### Chemotaxis Assays.

Chemotaxis and OL (also called olfactory adaptation) assays were performed as described by Colbert and Bargmann (16) with modifications by Bettinger and McIntire (6), and detailed in *SI Appendix, SI Materials and Methods*. Briefly, chemotaxis plates were prepared and allowed to dry overnight at room temperature. Immediately before the experiment, plates were dried without lids at 37 °C for 1 h. We added 100% ethanol to plates to yield 150 mM or 300 mM ethanol. Then 1 µL of diluted benzaldehyde (1:200 benzaldehyde to ethanol) was pipetted onto a spot on one side of the plate and 1 µL of diluent (100% ethanol) was pipetted onto a spot exactly opposite. To each spot, 1 μL of 1 M sodium azide was added to immobilize worms once they reached the spot. Between 50 and 100 worms were pipetted in 10 µL of assay buffer onto each plate at a position equidistant from the odorant and diluent spots and slightly off-center (Fig. 1*A*). After 1 h at room temperature, worms were counted and a chemotaxis index was calculated, as follows: chemotaxis index = (the number of worms at the odorant spot − the number of worms at the diluent spot) / the total number of worms on the plate.

### OL Assays.

Briefly, pre-exposure plates were prepared and allowed to dry overnight at room temperature. Immediately before the experiment, plates were dried without lids at 37 °C for 1 h. For ethanol-containing plates, 100% ethanol was added to plates to yield 150 mM or 300 mM ethanol. For benzaldehyde pre-exposure, 1 µL of 100% benzaldehyde was pipetted onto each of five solidified agar drops on the lids of the pre-exposure plates. Animals were washed off culture plates, then the single population was divided so that roughly equal numbers of animals were placed on all pre-exposure plates, which were then sealed with Parafilm. Animals were incubated in all pre-exposure conditions for 90 min at room temperature, then washed off plates, and then each pre-exposure population was divided in half and transferred to the paired chemotaxis plates for the chemotaxis assay.

### Optogenetics Methods.

All animals used in these studies were reared on OP50 bacteria that were supplemented 24 h before the culture was used for seeding bacterial lawns with 10 μM ATR (Sigma-Aldrich) except for animals that were used as non-ATR controls. Animals reared on ATR-supplemented bacteria were kept in the dark. All optogenetic experiments were performed in a dark room with a red light source to minimize any possible activation of channelrhodopsin by ambient light. To activate channelrhodopsin and anionic channelrhodopsin, we used a delivery paradigm described by Crawford and San-Miguel (62). For details, see *SI Appendix, SI Materials and Methods*.

### Data Analysis.

Unpaired, two-tailed, multiple *t* test analyses (false discovery rate, 5%) (Prism 9; Graphpad Software) were used to compare the mean chemotaxis indices of 0 mM vs. 150 mM ethanol-exposed chemotaxis assays unless otherwise noted. The multiple *t* test analyses take into account multiple testing in determining statistical significance; adjusted *P* values are presented.

## Supplementary Material

Supplementary File

## Data Availability

All study data are included in the article and/or supporting information.
