# Supplementary material for: A neuropeptide signal confers ethanol state dependency during olfactory learning in Caenorhabditis elegans
Source: Proc Natl Acad Sci U S A. 2022 Nov 7;119(46):e2210462119. doi: 10.1073/pnas.2210462119 (PMC9674237; doi:10.1073/pnas.2210462119)
Supplement: Supplementary File [file pnas.2210462119.sapp.pdf]

Supplementary Information for

A neuropeptide signal confers ethanol state-dependency during olfactory learning in *C. elegans.*

Jonathan H. Lindsay, Laura D. Mathies, Andrew G. Davies, Jill C. Bettinger

Jill C. Bettinger
Email: jcbettinger@vcu.edu

This PDF file includes:

Supplementary text
Figures S1 to S2
Table S1
SI References

SI Materials and Methods

***C. elegans* strains and husbandry.** *C. elegans* strains were maintained on lawns of *E. coli* strain OP50 on Nematode Growth Medium (NGM) at 20°C (1). Strains used were derived from wild-type Bristol N2 and were obtained from the *Caenorhabditis* Genetics Center, the National BioResource Project, or generated as described below. Strains used in this study were provided by the Caenorhabditis Genetics Center, which is funded by National Institutes of Health (NIH) Office of Research Infrastructure Programs Grant P40 OD010440.

Strains tested in this study:

N2
JC2154: *hen-1(tm501)* (2)
JT249: *scd-2(sa249)* (3)
OH8585: *otIs4[gcy-7::GFP]*; *otEx3822[ceh-36::CZ-caspase3(p17) + gcy-7::caspase3(p12)-NZ + myo-3::mCherry]* (4)
OH8593: *ntIs1[gcy-5p::GFP + lin-15(+)] V; otEx3830[ceh-36::CZ-caspase3(p17) + gcy-5::caspase3(p12)-NZ + myo-3::mCherry]* (4)
BZ142: *slo-1(eg142)* (5)

Transgenic lines created for this study:

JCB287: *hen-1(tm501); betEx3 [Pgcy-5::GFP::T2A::hen-1]*
JCB300: *scd-2(sa249); betEx8 [Pgcy-28.d::GFP::T2A::scd-2]*
JCB314: +/+; *betEx12 [Pgcy-5::ChR2(H134R)::YFP]*
JCB328: *hen-1(tm501); betEx12 [Pgcy-5::ChR2(H134R)::YFP]*
JCB340: *cat-2(e1112); betEx12 [Pgcy-5::ChR2(H134R)::YFP]*
JCB350: +/+; *betEx14 [Pgcy-5::GtACR1::tdTomato]*

All animals used in behavioral assays were age-matched first day adult hermaphrodites that had been reared for at least two generations in uncrowded, well-fed conditions. Three days prior to testing, populations of animals were age-matched as follows: 5 adult worms were allowed to lay eggs for 3-4 hours on each of 20 seeded 6 cm NGM culture plates, and then removed. The resulting progeny were allowed to develop and were tested on their first day of adulthood. In animals carrying constructs expressing channelrhodopsin or anionic channelrhodopsin, the culture plates were wrapped in aluminum foil to minimize possible effects of ambient light. For these strains, on the day before the SDL assay, L4-stage progeny were transferred to ATR-containing plates in a light-restricted room.

Chemotaxis Assays. Chemotaxis and olfactory learning (OL, also called olfactory adaptation) assays were performed as described by Colbert and Bargmann (6), with minor modifications as described by Bettinger and McIntire (7). Chemotaxis plates were prepared as follows: 10 mL of assay agar (2% agar, 5 mM KPO_4, 1 mM $MgSO_4$, 1 mM $CaCl_2$) was aliquoted into 10 cm petri plates and allowed to dry overnight at room temperature. Immediately before the experiment, plates were dried without lids at 37°C for 1 hour. Plates were weighed after drying and, for ethanol-containing plates, 100% ethanol was added to plates to yield 150 mM or 300 mM ethanol. The plates were sealed with Parafilm and ethanol was allowed to equilibrate into the agar for 1 hour. 1 µL of diluted benzaldehyde (1:200 benzaldehyde:ethanol) was pipetted onto a spot on one side of the plate and 1 µL diluent (100% ethanol) was pipetted onto a spot exactly opposite. To each spot, 1 µL of 1 M sodium azide was added to immobilize worms once they reached the spot. Age-matched first-day adult animals were added to plates after washing twice with S-basal (0.1 M NaCl, 0.05 M KPO_4[pH 6], 5 mg/l cholesterol) and once with assay buffer (5 mM KPO_4[pH 6], 1 mM $CaCl_2$, 1 mM $MgSO_4$). Between 50 and 100 washed worms were pipetted in 10 µL assay buffer onto each plate at a position equidistant from the odorant and diluent spots and slightly off-center (Figure 1A). Excess liquid was wicked off the plate using the corner of a Kimwipe. After one hour at room temperature, worms were counted; worms were considered to be at the odorant or diluent spots if they were within 1 cm of the spot. A chemotaxis index (CI) was calculated CI = (the number of worms at the odorant spot – the number of worms at the diluent spot)/the total number of worms on the plate. A high CI (close to 1) indicates the odorant acted as a strong attractant, whereas a lower CI indicates that the odorant was a less effective attractant.

C. elegans extrachromosomal arrays are subject to mosaic loss in each animal. We ensured that our cell-specific expression constructs were not lost from the relevant neurons in experimental animals by scoring chemotaxis assays under fluorescent illumination; only animals with the fluorescent marker were included in the calculation of the CI.

Olfactory Learning (OL) Assays. All OL assays were performed as described by Colbert and Bargmann (6) and modified as described in Bettinger and McIntire (7). Pre-exposure plates were prepared as follows: 10 mL of OL agar (3% agar, 5mM KPO_4, 1mM $MgSO_4$, 1mM $CaCl_2$) was aliquoted into 10 cm petri plates and allowed to dry overnight. Immediately before the experiment, plates were dried without lids at 37°C for 1 hour. Plates were weighed after drying and, for ethanol-containing plates, 100% ethanol was added to plates to yield 150 mM or 300 mM ethanol. The plates were sealed with Parafilm and ethanol was allowed to equilibrate into the agar for 1 hour. For benzaldehyde pre-exposure, 1uL of 100% benzaldehyde was pipetted onto each of five solidified agar drops on the lid of the pre-exposure plates. Animals were washed off culture plates with S-Basal, (0.1 M NaCl, 0.05 M KPO_4[pH6], 5 mg/l cholesterol) then washed once with S-basal and once with assay buffer (5 mM KPO_4[pH 6], 1 mM $CaCl_2$, 1 mM $MgSO_4$), then the single population was divided so that roughly equal numbers of animals were placed on all pre-exposure plates, which were then sealed with Parafilm. Animals were incubated in all pre-exposure conditions for 90 min at room temperature, then washed off plates with S-basal, washed once with S-basal, once with assay buffer, and then each pre-exposure population was divided in half and transferred to the paired chemotaxis plates (*i.e.* ethanol and no-ethanol or blue light exposure and no blue light exposure) for the chemotaxis assay. All conditions that were compared were

performed at the same time such that a single population of worms was divided when placed onto the pre-exposure plates.

Constructs. All primers and sequences used in this study can be found in Table S1. PCR was performed with the Q5 High Fidelity DNA Polymerase (New England Biolabs), DNA sequences were assembled using HIFI DNA Assembly Kit (New England Biolabs), or were generated using BsmI, BamHI, HindIII, or EagI restriction enzymes and ligated using a DNA ligation kit (ThermoFisher). The resulting clones were sequenced to ensure that no mutations were introduced. Extrachromosomal arrays carrying each DNA construct were generated through germline injection and transformation of an appropriate strain (8) and lines with a high transmission frequency for the extrachromosomal arrays were selected for analysis.

pPD95.75 T2A. We inserted T2A (9) downstream of GFP in pPD95.75 (Addgene plasmid 19327). T2A is a self-cleaving peptide that induces ribosome skipping during translation. The T2A sequence was synthesized as a gBlock (IDT) that included vector homology arms, eliminated the stop codon from GFP, and added an NheI site downstream of T2A. pPD95.75 was digested with BsmI and EcoRI and T2A was inserted using DNA assembly.

***hen-1* rescue.** The *gcy-5* promoter and *hen-1* coding sequences were amplified from N2 genomic DNA and assembled with GFP::T2A to create P*gcy-5::GFP::T2A::hen-1*. 3000 bp upstream of the *gcy-5* start codon was amplified using primers gcy-5p_Ffix and gcy-5p_Rfix. 792 bp of *hen-1* genomic sequence, including all exons and the 3' UTR, was amplified using primers hen-1_Ffix and hen-1_Rfix. pPD95.75 T2A was digested with HindIII and EcoRI to produce the vector backbone and GFP::T2A, which were combined with the *gcy-5* promoter and *hen-1* coding sequence using DNA assembly. The assembled product was injected into JC2154: *hen-1*(*tm501*) at 10 ng/µL with 10 ng/µL pCFJ90 (Addgene plasmid 19327) co-injection marker and 75 ng/µL 1 kb DNA ladder (New England Biolabs) to create the extrachromosomal array-bearing strain JCB287.

***scd-2* rescue.** The *gcy-28d* promoter and *scd-2* genomic sequence including all exons and the 3' UTR was amplified from N2 genomic DNA. First, 2841 bp upstream of the *gcy-28.d* promoter was amplified using primers gcy-28d_F and gcy-28d_R. pPD95.75 T2A was digested with HindIII and the *gcy-28.d* promoter was cloned upstream of GFP::T2A using DNA assembly to create *Pgcy-28.d::GFP::T2A*. Next, 6397 bp of *scd-2* genomic sequence, including all exons and 3' UTR, was amplified in two fragments using primers scd-2N_F and scd-2N_R and scd-2C_F and scd-2C_R. P*gcy-28.d::GFP::T2A* was amplified using primers vector_F and vector_R and all three fragments were assembled to create P*gcy-28.d::GFP::T2A::scd-2*. This plasmid was injected into JT249 *scd-2*(*sa249*) at 10 ng/µL with 10 ng/µL pCFJ90 (Addgene plasmid 19327) co-injection marker and 75 ng/µL 1 kb DNA ladder (New England Biolabs) to create the extrachromosomal array-bearing strain JCB300.

Channelrhodopsin-2 (ChR2) expression: We used a variant of channelrhodopsin ChR2(H134R) that has been reported to activate under low light intensity and sustain changes in behavior over long periods (10). 2560 bp of *ChR2::YFP* DNA sequence, from the ChR2 start codon to the end of the *unc-54* 3' UTR was PCR amplified from genomic DNA from the strain ZX299 bearing *zxEx22 [myo-3p::ChR2(H134R)::YFP + lin-15(+)]* (11) using chr2_F and chr2_R primers. The product was ligated in place of *GFP::T2A::hen-1* in the *hen-1* rescue construct using T4 DNA ligase (New England Biolabs) via engineered BamHI and EagI sites. The resulting *Pgcy-5::ChR2(H134R)::YFP* expression plasmid was injected into N2 worms at 10 ng/µL with 10 ng/µL pCFJ90 (Addgene plasmid 19327) co-injection marker and 75 ng/µL 1 kb DNA ladder (New England Biolabs) to create the extrachromosomal array-bearing strain JCB314: *betEx12* [*Pgcy-5::ChR2(H134R)::YFP*]. *betEx12* was crossed into the *hen-1(tm501)* background to create JCB328 and into the *cat-2*(*e1112*) background to create JCB340.

***Guillardia theta* Anion Channelrhodopsin-1 (GtACR1) expression:** The 2367 bp *GtACR1::tdTomato* DNA sequence was PCR amplified from pTol1-UAS:GtACR1-tdTomato

(Addgene plasmid 124235; 12) using GtACR1_fwd and GtACR1_rev primers. The 905 bp *unc-54* 3' UTR DNA sequence was PCR amplified from the P*gcy5::ChR2(H134R)::YFP* plasmid. P*gcy-5::ChR2(H134R)::YFP* was digested with ApaI and SalI to liberate the vector backbone and P*gcy-5* promoter; all three fragments were assembled by DNA assembly. The resulting *Pgcy-5::GtACR1::tdTomato* expression plasmid was injected into N2 worms at 10 ng/µL with 10 ng/µL pCFJ68 (Addgene plasmid 19325) coinjection marker and 75 ng/µL 1 kb DNA ladder (New England Biolabs) to create the extrachromosomal array-bearing strain JCB350: *betEx14* [*Pgcy-5::GtACR1::tdTomato]*.

Optogenetics methods. All animals used in these studies were reared on OP50 bacteria that were supplemented with 10 µM all-trans retinol (ATR; Sigma-Aldrich) 24 hours before the culture was used for seeding bacterial lawns, except for animals that were used as -ATR controls. Animals reared on ATR-supplemented bacteria were kept in the dark. All optogenetic experiments were performed in a dark room with a red light source to minimize any possible activation of channelrhodopsin by ambient light.

To activate channelrhodopsin ChR2, we used a delivery paradigm described by Crawford and San-Miguel (13): exposure consisted of 1-minute bouts made of 30 seconds of 465 nm light flashes [10 milliseconds on; 10 milliseconds off] and 30 seconds of no light; the number of minutes of light exposure varied in the different experiments and is described. We used Matlab (MathWorks) to drive an Arduino circuit board (Arduino) connected to an LED driver (Digikey) which held the current at 350mA; a single pole single throw (SPST) relay closed the circuit to allow a 1.5V LED (Digikey) to 'blink on' and 'blink off'. We measured light intensity at the center of the plate at 0.55 mw/mm^2 and minimally at 0.15 mw/mm^2 at the edge of the plate using a laser power meter (Sanwa).

The anionic channelrhodopsin GtACR1 is sensitive to green light that is in the 460-560 nm wavelength range. Because there are similar effects of GtACR1 on *C. elegans* behavior at 460 nm and 525nm light (14), we used the same light (465 nm) delivery protocol as described for channelrhodopsin ChR2.

Dopamine supplementation. Dopamine culture plates were prepared by adding dopamine solution (50 mM dopamine hydrochloride (DA; Sigma-Aldrich) in assay buffer) to a final concentration of 2 mM DA to ATR plates seeded with OP50. JCB340: *cat-2(e1112); betEx12 [Pgcy-5::ChR2(H134R)::YFP]* animals were cultured on these plates for 24 hours before testing as described for optogenetics experiments. For the SDL assay, dopamine was supplemented on pre-exposure plates (2 mM DA, 0.1% metabisulfite in assay buffer). We found that adding DA to the chemotaxis plates caused the animals to stop moving, confounding our ability to score the chemosensory response, and therefore we did not supplement DA during chemotaxis.

Fluorescent microscopy. Transgenic strains with fluorescent reporters were imaged using a Zeiss Axio Imager A1 microscope outfitted with a 63x Plan Apochromat objective and DIC optics (Zeiss). Images were captured with a Zeiss AxioCam MRm using Zen software (Zeiss).

Data analysis. Unpaired two-tailed multiple T-test analyses (FDR = 5%) (Prism 9, Graphpad Software) were used to compare the mean CIs of 0 mM vs. 150 mM ethanol exposed chemotaxis assays unless otherwise noted. The multiple T-test analyses take into account multiple testing in determining statistical significance; adjusted *p* values are presented.

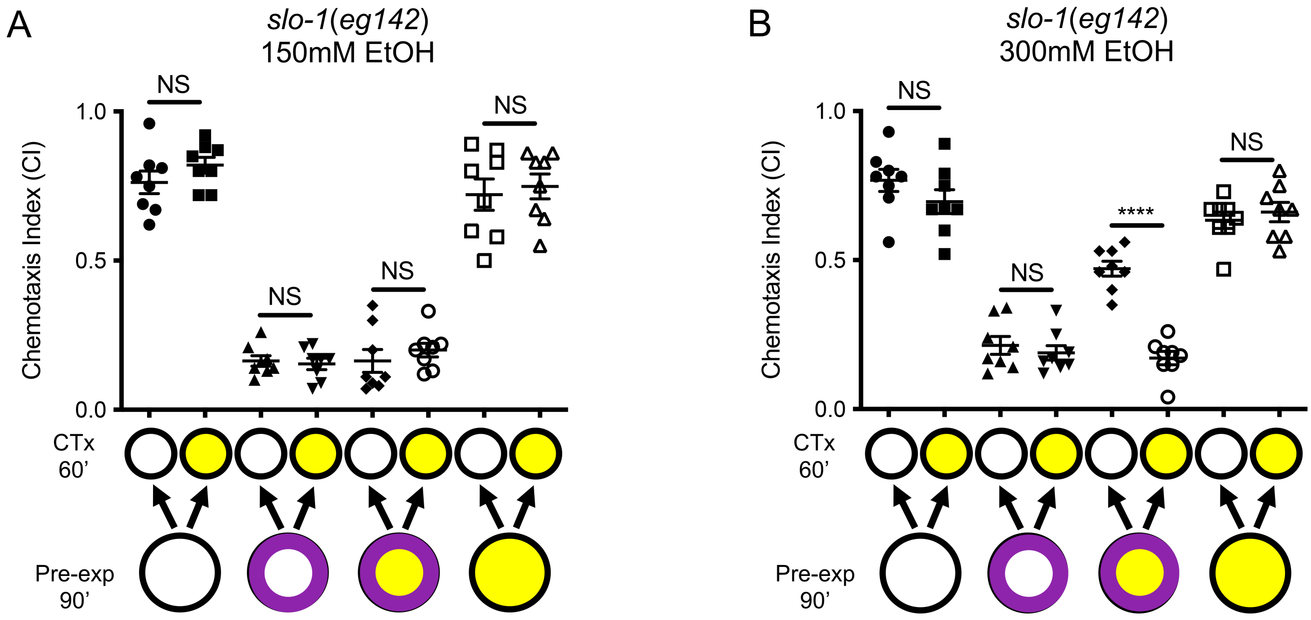
A
slo-1(eg142)
150mM EtOH
NS
NS
NS
NS
Chemotaxis Index (CI)
1.0
0.5
0.0
CTx
60'
Pre-exp
90'
B
slo-1(eg142)
300mM EtOH
NS
NS
****
NS
Chemotaxis Index (CI)
1.0
0.5
0.0
CTx
60'
Pre-exp
90'

Figure S1. ***slo-1*** **is required for SDL at 150 mM ethanol but not 300 mM ethanol.** (*A*) *slo-1(eg142)* null mutant animals do not learn state-dependently when trained and tested on 150 mM ethanol; OL that is learned during ethanol exposure is not dependent on ethanol exposure during testing (compare to N2 results in Fig. 1B). (*B*) In contrast, when *slo-1(eg142)* mutant animals are trained on 300 mM ethanol, expression of OL requires the presence of 300 mM ethanol during testing. Pale blue filled circle indicates blue light exposure; purple ring indicates benzaldehyde pre-exposure; yellow indicates ethanol exposure. Error bars represent SEM. Statistical comparisons were made using unpaired multiple T-tests (n=8); bars indicate which data sets are being compared; **** p<0.0001; NS not significantly different.

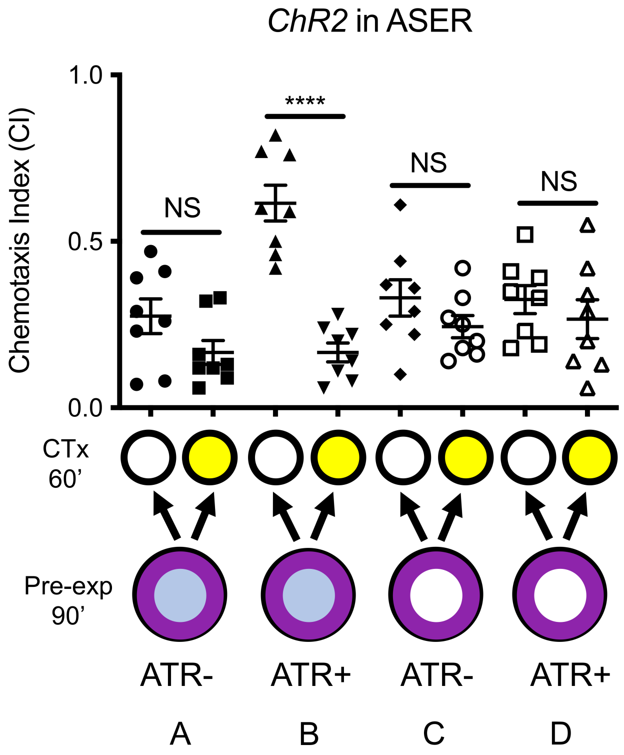

ChR2 in ASER
Chemotaxis Index (CI)
1.0
0.5
0.0
****
NS
NS
NS
CTx
60’
Pre-exp
90’
ATR-
ATR+
ATR-
ATR+
A
B
C
D

Figure S2. Activation of channelrhodopsin requires all-trans retinol (ATR). *betEx12* [*Pgcy-5::ChR2::YFP*] animals express channelrhodopsin in ASER. Animals were cultured in the presence of (+ATR) or absence of (-ATR) all-trans retinol for 24 hours before the experiment. Animals were pre-exposed to benzaldehyde for 90 min to induce AOL. AOL was observed by testing the chemotaxis response to benzaldehyde (CTx 60'). A low CI (below 0.5) indicates that the animals have undergone AOL and recall their pre-exposure to benzaldehyde. If AOL is state-dependent, demonstration of AOL requires the presence of ethanol during chemotaxis. (A) In animals reared without ATR, blue light during pre-exposure to benzaldehyde does not affect the expression of AOL; there is no evidence of state-dependency. (B) In contrast, in animals reared on ATR, optogenetic activation of ASER during pre-exposure to benzaldehyde confers state-dependency to AOL such that the expression of AOL is dependent on ethanol exposure during chemotaxis. (C, D) ATR exposure has no effect on AOL in the absence of blue light stimulation. Pale blue filled circles represent blue light exposure; purple ring indicates benzaldehyde pre-exposure; yellow indicates ethanol exposure. Error bars represent SEM. Statistical comparisons were made using unpaired multiple T-tests (n=8); bars indicate which data sets are being compared; **** q<0.0001; NS not significantly different.

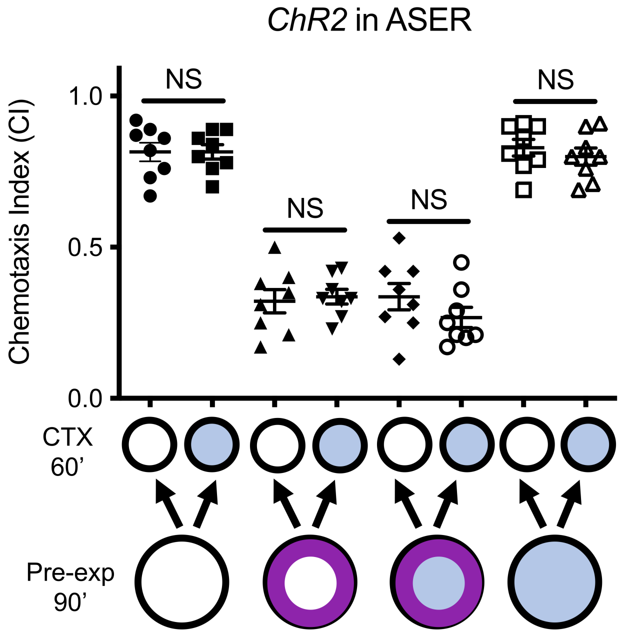

ChR2 in ASER
Chemotaxis Index (CI)
1.0
0.5
0.0
NS
NS
NS
NS
CTX
60'
Pre-exp
90'

Figure S3. Optogenetic activation of the ASER neuron during both training and testing does not substitute for ethanol to generate SDL. Optogenetic activation of ASER in *betEx12* [*Pgcy-5::ChR2::YFP*] animals during both pre-exposure and testing, in the absence of ethanol, does not replace ethanol exposure in SDL. Pale blue filled circles represent blue light exposure; purple ring indicates benzaldehyde pre-exposure; yellow indicates ethanol exposure. Error bars represent SEM. Statistical comparisons were made using unpaired multiple T-tests (n=8); bars indicate which data sets are being compared; NS not significantly different.

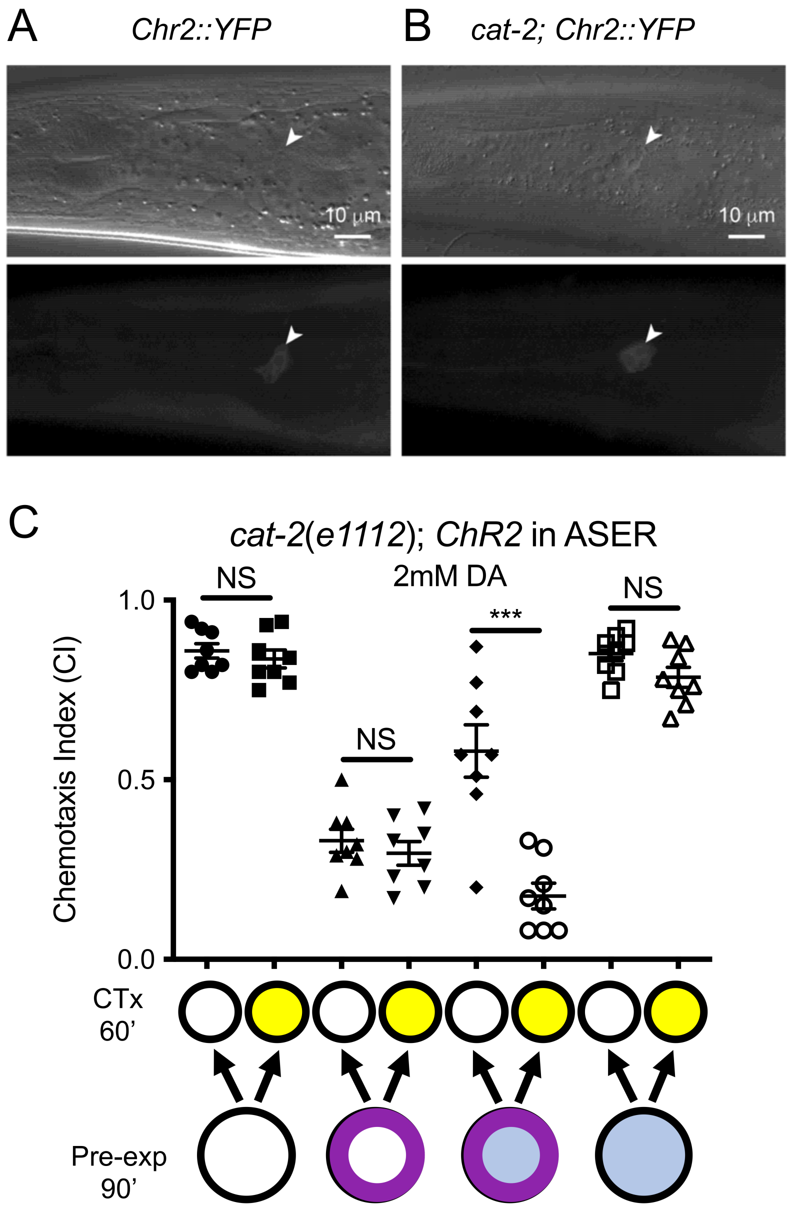
A
Chr2::YFP
B
cat-2; Chr2::YFP
10 μm
10 μm
C
cat-2(e1112); ChR2 in ASER
2mM DA
NS
NS
***
NS
1.0
0.5
0.0
Chemotaxis Index (CI)
CTx
60'
Pre-exp
90'

Figure S4: ASER activation can cause SDL in *cat-2* mutants supplemented with exogenous dopamine. *(A)* YFP expression in a young adult +/+; *betEx12 [Pgcy-5::ChR2(H134R)::YFP]* animal (top) fluorescent image (exposure 500 ms; white arrowhead indicates ASER) (bottom) DIC image (exposure 50 ms) Anterior is to the left; scale bar, 10 μm. *(B)* YFP expression in a young adult *cat-2(e1112); betEx12 [Pgcy-5::ChR2(H134R)::YFP]* animal. (top) fluorescent image (exposure 500 ms; white arrowhead indicates ASER) (bottom) DIC image (exposure 50 ms). Anterior is to the left; scale bar, 10 μm. *(C)* Supplementation of 2 mM exogenous dopamine can restore the ability of activation of ASER to confer state-dependency to OL in *cat-2(e1112); betEx12 [Pgcy-5::ChR2(H134R)::YFP]* animals (compare to Fig. 6). Pale blue filled circles represent blue light exposure; purple ring indicates benzaldehyde pre-exposure; yellow indicates ethanol exposure. Error bars represent SEM. Statistical comparisons were made using unpaired multiple T-tests (n=8); bars indicate which data sets are being compared; *** q<0.0001; NS not significantly different.

Table S1. Synthetic sequences and primers used in this study.

Sequence name	Sequence
pPD-nT2A	5' -ctgggattacacatggcatggatgaactatacaaaga gggcagaggaagtctgctaacatgcggtgacgtcgagga gaatcctggcccagaattccaactggctagcgccggtcgc taccattaccaacttgtctggtg – 3'

Primer name	Sequence
hen-1_F	5' – aggagaatcctggcccaATGAACTCCAAAGTAATTTTTTTCgtatg – 3'
hen-1_R	5' – gaccggcgctcagttggaattgtgtttacaaaattgtttatt – 3'
gcy-5p_F	5' – caacttggaaatgaaataTTTCCACCATTTGAGCAAG – 3'
gcy-5p_R	5' – ctgcaggcatgcaagctTTTTCATCAGAATAAGTAATTTTTCG – 3'
gcy28p_F	5' – gctaacaacttggaaatgaaataTACAATTGTAGTGAGCTTCG – 3'
gcy28p_R	5' – cgacctgcaggcatgcaagctTTCGCACTCATCTCACCATTC – 3'
scd-2N_F	5' – cctggcccaATGCGTAAACGAAGACTATGGTGG – 3'
scd-2N_R	5' – CGAAGCTCATCATCGTGCTC – 3'
scd-2C_F	5' – TCACGGAGCACGATGATGAG – 3'
scd-2C_R	5' – cgactagtaGAAAATACATGGCATTTATTCACAAGG – 3'
vector_F	5' – atgccatgtattttCTACTAGTCGGCCGTACGG – 3'
vector_R	5' – CTTCGTTTACGCATtgggccaggattctcctcg – 3'
chr2_assemble_F	5' – GCAGGTCGACTCTAGAGGATCCATGGATTATGGAGGC – 3'
chr2_assemble_R	5' – CGAGACGAAAGGGCCCGTACGGCCGACTAGTAGGAAAC – 3'.
GtACR1_F	5' – aaagcttgcatgcctgcaggatgagcagcatcacctgtg – 3'
GtACR1_R	5' – gttggaattcttacttatacagctcatccatgc – 3'
unc_54_UTR_F	5' – gtataagtaagaattccaactgagcgccgg – 3'
unc_54_UTR_R	5' – acgcgcgagacgaaagggcc – 3'

SI References

1. S. Brenner, The genetics of *Caenorhabditis elegans*. *Genetics* **77**, 71–94 (1974).
2. T. Ishihara, *et al.*, HEN-1, a secretory protein with an LDL receptor motif, regulates sensory integration and learning in Caenorhabditis elegans. *Cell* **109**, 639–649 (2002).
3. T. Inoue, J. H. Thomas, Suppressors of transforming growth factor-β pathway mutants in the Caenorhabditis elegans dauer formation pathway. *Genetics* **156**, 1035–1046 (2000).
4. C. O. Ortiz, *et al.*, Lateralized Gustatory Behavior of *C. elegans* Is Controlled by Specific Receptor-Type Guanylyl Cyclases. *Curr. Biol.* **19**, 996–1004 (2009).
5. A. G. Davies, *et al.*, A central role of the BK potassium channel in behavioral responses to ethanol in *C. elegans*. *Cell* **115**, 655–666 (2003).
6. H. A. Colbert, C. I. Bargmann, Odorant-specific adaptation pathways generate olfactory plasticity in *C. elegans*. *Neuron* **14**, 803–812 (1995).
7. J. C. Bettinger, S. L. McIntire, State-dependency in *C. elegans*. *Genes, Brain Behav.* **3**, 266–272 (2004).
8. C. Mello, A. Fire, DNA Transformation. *Methods Cell Biol.* **48**, 451–482 (1995).
9. A. Ahier, S. Jarriault, Simultaneous expression of multiple proteins under a single promoter in *Caenorhabditis elegans* via a versatile 2A-based toolkit. *Genetics* **196**, 605–613 (2014).
10. C. Schultheis, J. F. Liewald, E. Bamberg, G. Nagel, A. Gottschalk, Optogenetic long-term manipulation of behavior and animal development. *PLoS One* **6**, e18766 (2011).
11. G. Nagel, *et al.*, Light activation of Channelrhodopsin-2 in excitable cells of *Caenorhabditis elegans* triggers rapid behavioral responses. *Curr. Biol.* **15**, 2279–2284 (2005).
12. P. Antinucci, *et al.*, A calibrated optogenetic toolbox of stable zebrafish opsin lines. *Elife* **9** (2020).
13. Z. Crawford, A. San-Miguel, An inexpensive programmable optogenetic platform for controlled neuronal activation regimens in *C. elegans*. *APL Bioeng.* **4**, 16101 (2020).
14. T. Yamanashi, *et al.*, Quantitation of the neural silencing activity of anion channelrhodopsins in *Caenorhabditis elegans* and their applicability for long-term illumination. *Sci. Rep.* **9** (2019).
